# ﻿A new species of *Hypothyce* (Coleoptera, Scarabaeidae, Melolonthinae) from Alabama, United States of America

**DOI:** 10.3897/zookeys.1168.104272

**Published:** 2023-06-23

**Authors:** Joe A. MacGown, JoVonn G. Hill

**Affiliations:** 1 Mississippi Entomological Museum, Mississippi State University, Starkville, Mississippi State, MS 39762, USA Mississippi State University Starkville United States of America

**Keywords:** Coastal Plain, endemic, identification key, sandhills, scarab, taxonomy

## Abstract

A new species, *Hypothycerayi* MacGown & Hill, **sp. nov.** (Coleoptera: Scarabaeidae: Melolonthinae: Melolonthini), is described from east central Alabama, USA. Three other species of *Hypothyce*, *H.burnei* Skelley, *H.mixta* Howden and *H.osburni* (Cartwright) are known to occur in the United States. We discuss differences between these species and provide an updated identification key to the genus.

## ﻿Introduction

Three species of *Hypothyce* have been reported from North America including *H.mixta* Howden, only known from Texas; *H.burnei* Skelley, only reported from Georgia; and *H.osburni* (Cartwright), only known from Georgia. Here, we describe *H.rayi* MacGown & Hill, sp. nov., known from one site in east central Alabama, and we provide an updated identification key to the genus.

**Figure 1.**
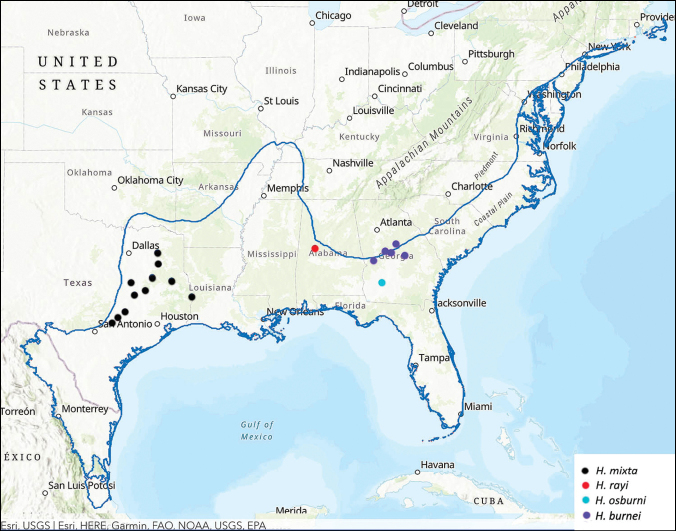
Distribution map of *Hypothyce* showing the localities of *H.mixta*, *H.burnei*, *H.osburni* and *H.rayi*. The blue line indicates the boundary of the North American Coastal Plain.

All of the described species of *Hypothyce* have been reported to inhabit isolated sandhills along the North American Coastal Plain (Barfield and Gibson 1975; Riley and Wolfe 2003; Skelley 2003), an area recently designated as the world’s 36^th^ global biodiversity hotspot based on the high levels of biodiversity and endemism of vascular plants and habitat loss greater than 70% in the region (Noss 2016) (Fig. 1). Much of the biodiversity of the North American Coastal Plain is found in grassland communities, such as the sandhills where *Hypothyce* occurs, (Noss 2012; Noss et al. 2014, 2021; Hill and Barone 2018). Barfield and Gibson (1975) wrote that *H.mixta* occurred in “isolated, sandy areas which are sparsely covered with hardwood, pine, and herbaceous vegetation.” *Hypothyceburnei* was described by Skelley (2003) from specimens collected at a porch light in upland sandhill habitat in Monroe County, Georgia. Skelley (2003) listed other records of *H.burnei* from Bibb, Jones, and Wilkinson Counties, Georgia, with all of the specimens collected by beating dead pines on sandy hill tops. In 2019, K. Schnepp collected this species in flight interception traps in Fall Line sandhill habitat in Taylor County, Georgia (Fig. 1).

The only known specimens of *H.osburni* were collected with a blacklight trap at a single locality in a pecan orchard in Doughtery County, Georgia (Cartwright 1939) (Fig. 1). Although Cartwright did not include information about the beetles being in dune habitat, Skelley (2003) speculated that, based on *H.mixta* collections in Texas, *H.osburni* specimens were likely collected from relictual fossil dunes located along the Flint River. In 2018 (on three dates), Alan Jeon collected three male specimens of *Hypothyce* resting on vegetation at night in sandhill habitat in Hale Co., Alabama (Fig. 1). Upon closer examination, it was clear that his collections represented a new species of *Hypothyce*.

## ﻿Material and methods

Three male specimens of *H.rayi* were examined and compared to male specimens of *H.burnei* (three specimens) and *H.osburni* (one specimen) borrowed from the Florida Collection of Arthropods, Gainesville, Florida (**FSCA**). The holotype and one paratypes of *H.rayi* MacGown & Hill, sp. nov. were deposited in the Mississippi Entomological Museum (**MEM**) and one paratype will be deposited in the Florida State Collection of Arthropods (FSCA). Genitalia were dissected and examined in 95% ethanol. Photomicrographs were captured using a Leica DFC 495 digital camera mounted on a Leica Z16 microscope with motorized Z-stepping, and image stacks were merged using Leica Application Suite v. 4.1.0 with Montage Module. All images were edited in Photoshop CS6. Measurements were made using a reticule placed in a 10× eyepiece of a Leica MZ16 stereomicroscope at a magnification of 10–100×.

### ﻿Key to the species of *Hypothyce* in the United States

This key is based on couplet 6 from Skelley (2003) and modified to include the new species described here.

**Table:** 

1	Protibia distinctly tridentate; metatibial spurs longer than two basal tarsomeres; only known from Texas	***Hypothycemixta* Howden**
–	Protibia bidentate to slightly tridentate; metatibial spurs shorter than two basal tarsomeres; only known from Alabama and Georgia	**2**
2	Apical margin of clypeus truncate to slightly concave (Fig. 2A); only known from Flint River region near Albany, Georgia	***Hypothyceosburni* (Cartwright)**
–	Apical margin of clypeus strongly convex (Fig. 2B, C)	**3**
3	Length greater than 22 mm; pronotum and elytra with dense, fine pubescence that does not completely obscure the integument except at lateral edges and along midline (Fig. 3A); scutellum sharply edged, with dense overlapping, thickened, elongate, white setae, which almost reach lateral edges of scutellum (Fig. 4A); only known to occur in sandhill habitat in central Georgia	***Hypothyceburnei* Skelley**
–	Length less than 19 mm; pronotum and elytra with dense, thickened, lanceolate yellowish whitish setae that mostly obscures the integument giving it a yellowish-white cast (Fig. 3B); scutellum with blunt lateral edges with visible thickness, with lanceolate yellowish-white setae about twice as long as setae on elytra) that do not reach lateral edges of scutellum (Fig. 4B); only known from longleaf pine/sand ridge habitat in Hale County, Alabama	***Hypothycerayi* MacGown & Hill, sp. nov.**

## ﻿Taxonomy

### ﻿ Hypothyce rayi

Taxon classificationAnimaliaColeopteraScarabaeidae

MacGown & Hill sp. nov.

EA42AC56-0D88-56C9-B9FA-114EA0E66584

https://zoobank.org/51FB1EE9-27CF-4AF2-A2DA-2FC4DEFA3F93

**Figure 2.**
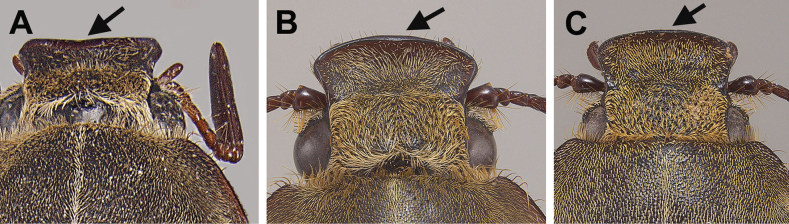
Dorsal view of clypei of **A***H.osburni* (arrow points to the slightly concave clypeal margin) and **B***H.burnei***C***H.rayi* (arrows point to the convex clypeal margin).

**Figure 3.**
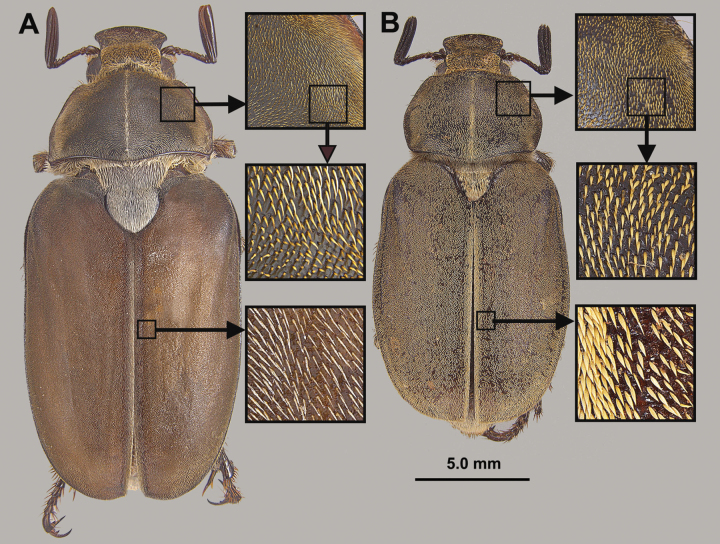
Dorsal view of **A***H.burnei* and **B***H.rayi* showing differences in setation.

**Figure 4.**
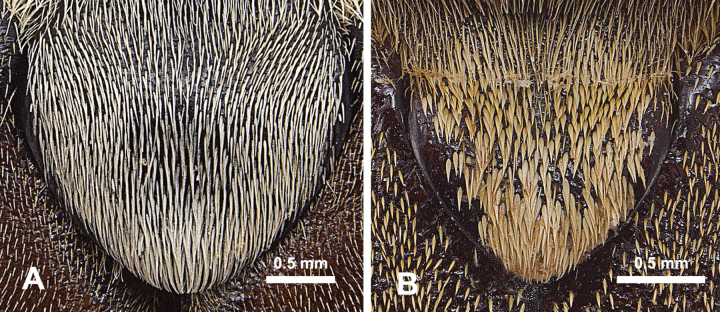
Scutella of **A***H.burnei* and **B***H.rayi* showing differences in setation.

**Figure 5.**
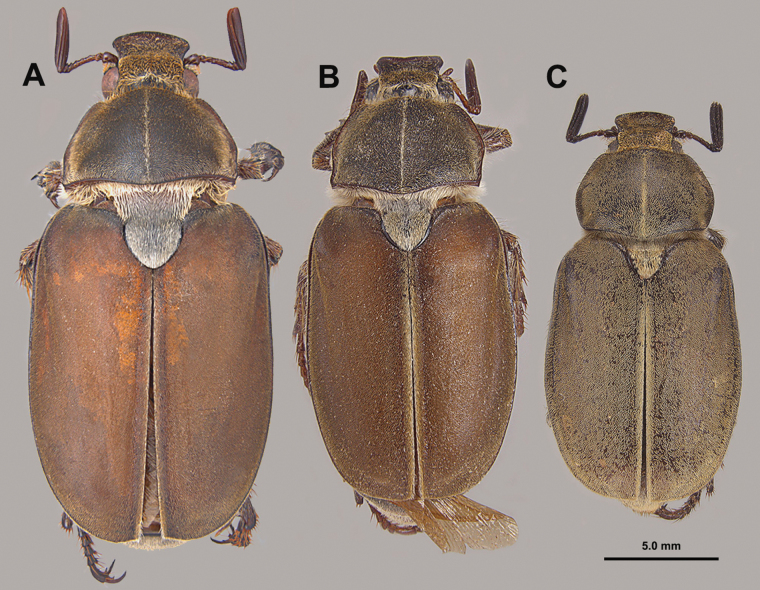
Dorsal habitus views of **A***H.burnei***B***H.osburni* and **C***H.rayi*.

**Figure 6.**
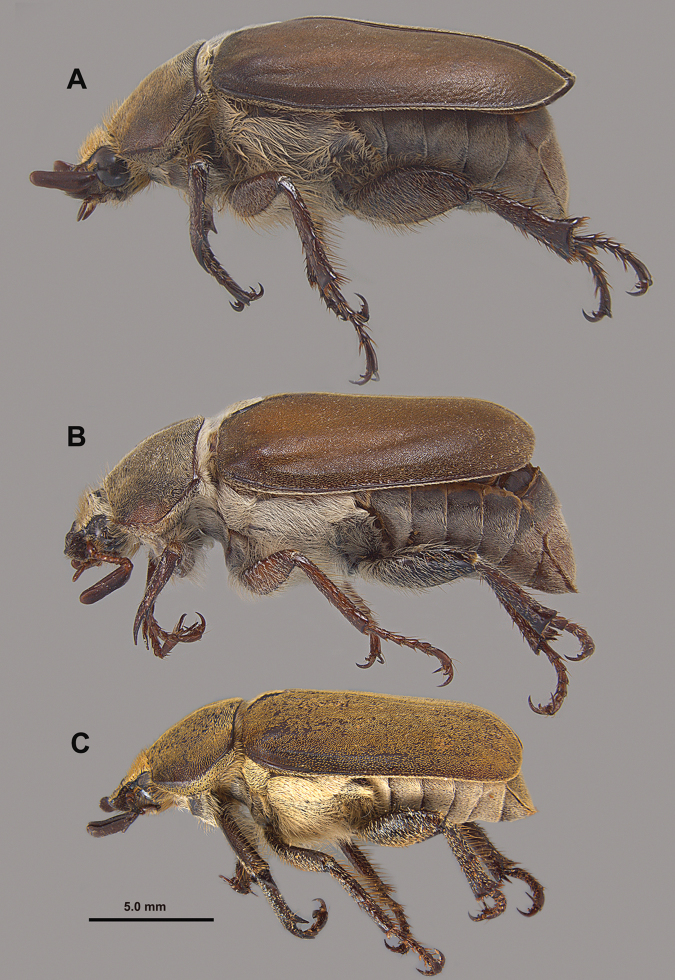
Lateral habitus views of **A***H.burnei***B***H.osburni* and **C***H.rayi*.

**Figure 7.**
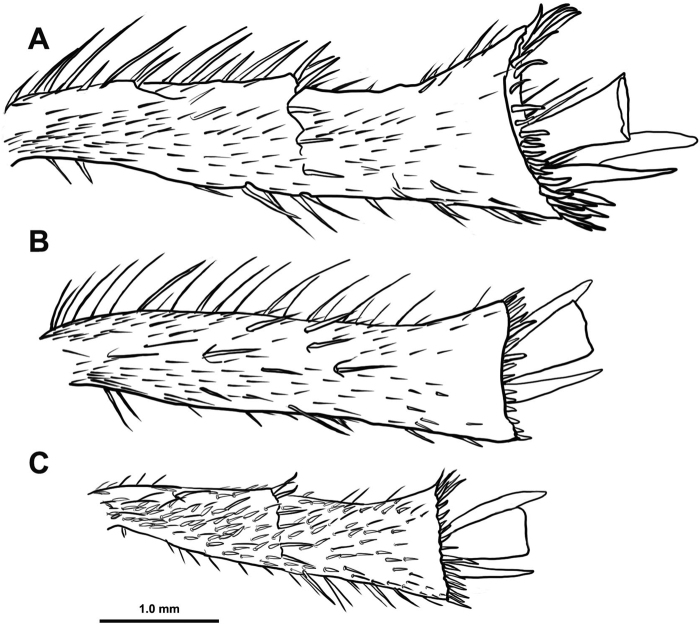
Metatibia of **A***H.burnei***B***H.osburni* and **C***H.rayi*.

**Figure 8.**
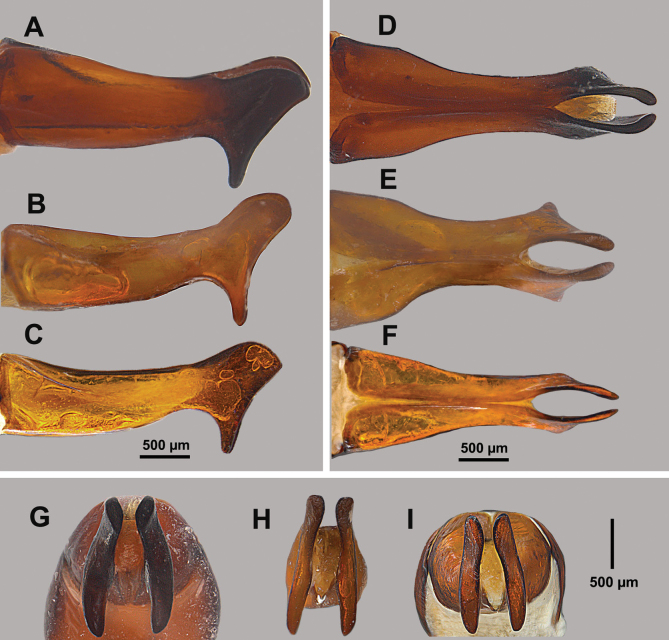
Male genitalia of *Hypothyce*: lateral view of **A***H.burnei***B***H.osburni* and **C***H.rayi*; dorsal view of **D***H.burnei***E***H.osburni* and **F***H.rayi*; apical view of **G***H.burnei***H***H.osburni* and **I***H.rayi*.

Figs 2C , 3B , 4B , 5C , 6C , 7C , 8C, F, I

#### Diagnosis.

Members of the genus *Hypothyce* can be distinguished from most Melolonthini (as defined by Evans and Smith 2020) by having an antennal club with three antennomeres; the antennae of most others in this tribe terminate in 5–7-antennomere clubs. *Hypotrichiaspissipes* LeConte also has a club with three antennomeres but can be differentiated from *Hypothyce* species by the accessory tooth on the anterior tarsal claw being lobe-like instead of small and acute as in of *Hypothyce* and by abdominal sutures between ventrites II–IV being distinct at midline, unlike in *Hypothyce* where sutures at midline are indistinct due to dense setation (Skelley 2003).

*Hypothycerayi* (Figs 5C, 6C) can be distinguished from other southeastern *Hypothyce* species by its generally smaller size (TL 17.3–18.7 mm as compared to 17.8–21 mm; based on a limited sample size, N = 3), pronotum and elytra with dense, lanceolate scale-like setae that mostly obscure the integument (Fig. 3B), femora and tibiae with thickened scale-like setae on outer faces, and by being only known from longleaf pine/sandhill habitat in Hale, County, Alabama. This species is only known from three male specimens; the female is unknown.

#### Description.

Holotype male. Total length 17.3 mm, width 8.5 mm (measured at widest part of elytra). Elongate oval; entire body dark reddish brown, integument mostly obscured by dense, appressed, short, yellowish-white, lanceolate setae; pronotum with a distinct median stripe of yellowish-white, lanceolate setae. Head width including eyes 3.3 mm, in dorsal view head including clypeus somewhat rectangular; clypeal width 2.6 mm, clypeal margin broadly rounded and slightly convex at middle, broadly and deeply recurved, trough-like, surface with dense, appressed, short (≈ 0.25 in length), whitish, lanceolate setae that arise from strong punctures, setae projecting inward or anteriorly, setae longer than those on pronotal and elytral dorsum (≈ 0.25 mm as compared to 0.10–0.20 mm); numerous erect, longer, narrower, setae that project posteriorly interspersed between appressed lanceolate setae. Eyes large, 1.5 mm at longest point, visible dorsally and ventrally, distance between eyes (in dorsal view) approximately four times eye length; eye partially bisected anteriorly by a clypeal projection. Antenna reddish brown; with 10 antennomeres including a three-antennomere, lamellate club, length of club subequal to length of antennomeres I–VII; scape (antennomere I) pyriform with stiff, elongate setae projecting apically and along ventral edge; pedicel (antennomere II) similar in shape to scape but lacking setae; antennomeres III–V slightly longer than wide, antennomeres III and V subequal, antennomere IV slightly longer, each with few stiff, elongate setae present medially; antennomeres VIII and IX cupped, wider at base and narrowing apically.

Pronotum wider than long (6.0 mm wide, 4.1 mm long), widest in middle, posterior angles rounded; with dense, appressed, yellowish-white, lanceolate setae, setae shorter (≈ 0.10 mm in length) than on head, denser medially forming a median stripe, less dense at sides of pronotum; rim of pronotum with narrow, upward curved setae present. Scutellum heart shaped, 1.5 mm in length and 1.5 mm at widest point; edges rounded; most of the surface with dense, overlapping, appressed, lanceolate, yellowish-white setae present except along the outer rim where setae are absent, setae longer, wider, and denser than those on elytra.

Elytra 11.0 mm in length, 8.5 mm wide; with strong lateral margins; area posterior of humeri toward outer edges of elytra slightly depressed; elytral surface with dense, appressed, short, yellowish-white, lanceolate setae that mostly obscure integument, setae similar in shape and length to those on pronotum (≈ 0.10 mm in length), setae denser along midline of elytra.

Sterna with dense, overlapping, elongate yellowish-white setae. Abdominal sternites and pygidium with short, appressed, whitish yellow, lanceolate setae that mostly obscure the integument but with margins visible and with a smooth shiny area medially.

***Legs***: Femora reddish brown, with dense, yellowish-white, elongate, curved setae and with numerous appressed, yellowish-white, scale-like setae on anterior and ventral surfaces. Protibia reddish brown, with two strong teeth and one smaller tooth located about one third from tibial base, apical tooth strongly curved; one tibial spur present; spine-like setae present along dorsal edge; numerous elongate, erect, curved, setae present ventrally and scattered on anterior surface; numerous appressed, yellowish-white, scale-like setae on anterior and ventral surfaces. Mesotibia reddish brown, lacking large, curved teeth, but with two partial ridges located about 1/3 and 2/3 from tibial base, extending from ventral edge and angling across anterior surface toward apex of tibia, each with a row of spine-like setae present; two tibial spurs present, one about 1.5 times as long as the other; tibia widened apically and rimmed with elongate spine-like setae; spine-like setae present along dorsal edge; numerous elongate, erect, curved, setae present ventrally and scattered on anterior surface; numerous appressed, and yellowish-white, scale-like setae on anterior surface. Metabia (Fig. 7C) similar to mesotibia except tibial spurs larger (≈ 0.10 and 0.07 mm), with longer spur widened and rounded apically, larger spur about the same length as metatarsus 1 and 2 combined. Five tarsal segments, each reddish brown, tarsus 1 ≈ 1.5 times as long as tarsi 2, 3, or 4, which are subequal in length, tarsus 5 approximately twice the length of tarsi 2, 3, or 4; tarsal claw with acute accessory tooth present, claws including accessory teeth with shallow longitudinal grooves present.

***Genitalia***: In lateral view parameres widened apically into a flattened club shape, upper part of club rounded rectangular and lower area in the form of a rounded triangle (Fig. 8C); in dorsal view parameres widest at base, narrowing apically before each side splits off at which point each paramere curves away from midline, forming a gently curved oval space between them, apical tips almost touching (Fig. 8C); in apical view each paramere appears elongate, about the same width from top to bottom, with each diverging from one another ventrally (Fig. 8I).

#### Variation.

Length 17.3–18.7 mm, width 8.5–8.8 mm (*N* = 3). Notable variation was not observed in setation or other physical characteristics.

#### Type material.

***Holotype***, male, “Hale Co., AL, 32.9964°, -87.4932°, July 16, 2018, coll: Alan Jeon, found resting on leaf in pine scrub habitat [MEM].” Two ***paratypes*** have the same data as the holotype except collections were made on July 8, 2018, and July 21, 2018 [MEM].

#### Etymology.

We named this species in honor of Dr. Charles Ray, an Alabama naturalist who recognized that this collection represented a new species.

#### Comments.

As with the other two species of eastern *Hypothyce*, *H.burnei* and *H.osburni*, little is known about the natural history of *H.rayi*. All three species appear to be restricted to sandhill habitats, each geographically separated from one another. Thus far, only males of the three eastern species have been collected, and they were capable of flight. Although the females for these three species are still unknown, Skelley (2003) speculated that *H.burnei* and *H.osburni* were flightless, and likewise, we believe that the females of *H.rayi* are flightless as well. This is based on the fact that females of related *H.mixta*, which have been found, were flightless (Barfield and Gibson 1975), and it would explain the apparent endemism shown by the three eastern species.

**Figure 9.**
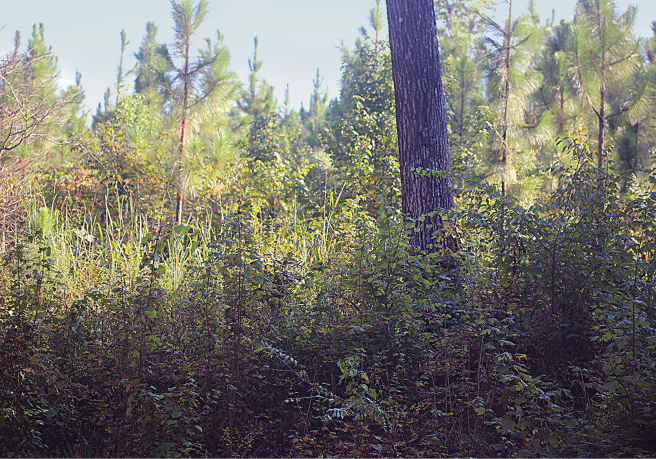
This photo, taken in the late summer of 2022, shows the thick understory now present at the type locality for *H.rayi* in Hale County, Alabama.

The type specimens of *H.rayi* were collected by Jeon in mid to late July on vegetation at night in what he described as scrub habitat in a longleaf forest. Charles Ray and MacGown made a return trip to the type locality in late August 2022, with several subsequent trips by Ray throughout the month, in hopes of finding additional specimens of *H.rayi*, especially females, which are unknown, but were unsuccessful in finding specimens. We believed that the habitat had changed significantly from 2018 when the beetles were collected, as the area now had a thick understory present (Fig. 9). The dense vegetation could be an unsuitable condition for this species (which has not been studied), and at the very least it made it difficult for us to search for the beetles. It is also likely that *H.rayi* has a limited time period when it is active, and that our search was simply too late in the year to find the beetle.

## Supplementary Material

XML Treatment for Hypothyce rayi

## ﻿Additional information

### Data availability

All of the data that support the findings of this study are available in the main text.
